# Supplementary material for: Towards unified AI-driven fracture mechanics: the extended deep energy method (XDEM)
Source: Nat Commun. 2026 Aug 15;17:8492. doi: 10.1038/s41467-026-76748-1 (PMC13484471; doi:10.1038/s41467-026-76748-1)
Supplement: Supplementary file 3 [file 41467_2026_76748_MOESM3_ESM.zip › pic/Results/Stress_intensity_factor/Cross/comparison_xdem_fem/angle_60/fem_dem_error_uv_3x2.pdf]

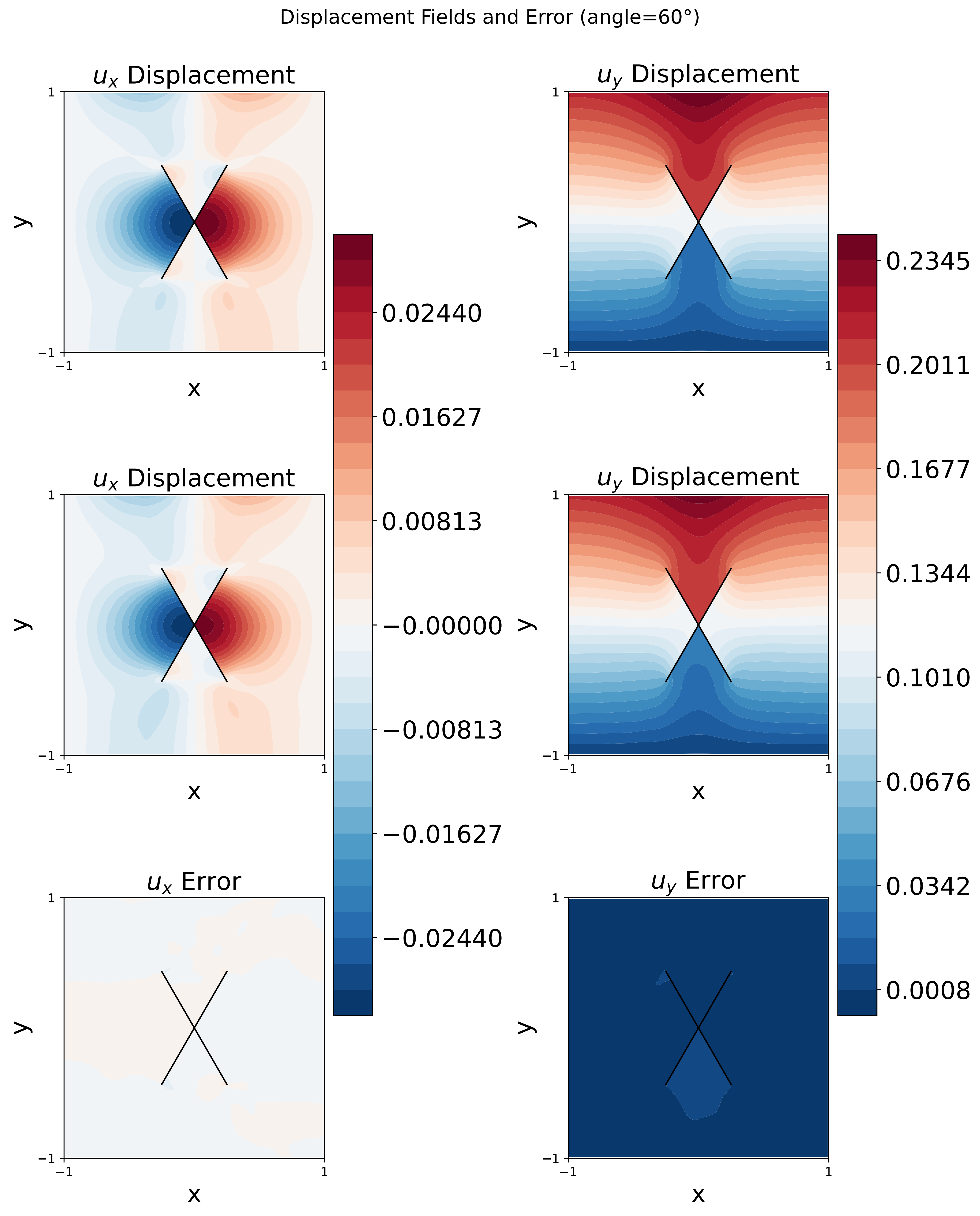
Displacement Fields and Error (angle=60°)
u_x Displacement
u_y Displacement
u_x Displacement
u_y Displacement
u_x Error
u_y Error
x
y
1
−1
0.02440
0.01627
0.00813
−0.00000
−0.00813
−0.01627
−0.02440
0.2345
0.2011
0.1677
0.1344
0.1010
0.0676
0.0342
0.0008
